# Voices From the Field: Stakeholder Perceptions Regarding the District Residency Program Implementation in the State of Kerala, India

**DOI:** 10.7759/cureus.94709

**Published:** 2025-10-16

**Authors:** Ravindran Chirukandath, Rajany Jose, Sumin V Sulaiman, Viswanathan K V, Ajithprasad J S, R Ashitha Menon, Keerthana Mohan, Reena K J, Thomas Mathew

**Affiliations:** 1 General Surgery, Government Medical College, Thrissur, Thrissur, IND; 2 Community Medicine, Government Medical College, Ernakulam, Ernakulam, IND; 3 General Surgery, Government Medical College, Trivandrum, Trivandrum, IND; 4 Physiology, Government Medical College, Thrissur, Thrissur, IND; 5 Public Health, Directorate of Health Service, Trivandrum, Trivandrum, IND; 6 Medical Education, Government Medical College, Trivandrum, Trivandrum, IND

**Keywords:** delphi method, district residency program (drp), health system strengthening, health workforce training, kerala, mixed-method approach, national medical council, postgraduate medical education, stakeholder perceptions

## Abstract

Background and rationale

The National Medical Council (NMC) introduced the District Residency Program (DRP) in 2020, mandating a three-month rotational residential work in district hospitals for postgraduate medical students with the aim of revamping the traditional postgraduate medical education system. Despite its potential benefits, the DRP has sparked debate among stakeholders. This study thus aims to evaluate the stakeholder perceptions and challenges in the implementation of the DRP in Kerala using a mixed-method approach. Unlike earlier evaluations from other Indian states, Kerala presents a unique context due to its early and comprehensive implementation of the DRP, strong public health infrastructure, and high stakeholder engagement, making it an ideal setting to generate transferable lessons for national-level policy refinement.

Materials and methods

The study was conducted at Government Medical College, Thrissur, Kerala, with participation from stakeholders across the state. A cross-sectional design was used for the quantitative part of the study, and the Delphi technique was used as a qualitative approach to identify perceptions of implementing officials and their proposed solutions to the issues identified. Informed consent was obtained from all participants before collecting information for the study. A total of 586 stakeholders participated in the study (residents n = 282; Directorate of Medical Education (DME) officials n = 180; Directorate of Health Services (DHS) officials n = 124). Quantitative data were analysed descriptively using IBM SPSS Statistics for Windows version 26, and qualitative insights were collected using a three-round Delphi process with 25 invited senior stakeholders (retention 88%).

Results

A total of 586 stakeholders participated (residents n = 282; DME officials n = 180; DHS officials n = 124). Most residents described district postings as providing diverse clinical exposure but identified gaps in infrastructure and supervision continuity. DME officials acknowledged the program’s positive impact on service delivery and training but noted the need for standardized evaluation systems. DHS officials reported improved patient care and staffing at peripheral hospitals, while emphasizing the importance of better accommodation and coordination mechanisms. The District Residency Programme (DRP) was perceived to have a positive impact on postgraduate training by enhancing residents’ clinical exposure, independent decision-making, and familiarity with peripheral health systems. However, gaps remained in infrastructure, supervision uniformity, and academic linkage between district hospitals and medical colleges.

Conclusion

The study highlights that while the DRP in Kerala has enhanced postgraduate training and strengthened district-level healthcare delivery, further progress requires investment in infrastructure, specialty-specific postings, and structured monitoring mechanisms to ensure uniform academic and service outcomes across sites.

## Introduction

In 2020, the National Medical Commission (NMC) launched the District Residency Program (DRP), requiring postgraduate medical residents to complete a three-month posting in district hospitals as part of their training [1]. This initiative was conceived as part of the broader shift towards competency-based medical education, aiming to strengthen clinical training by exposing residents to district-level healthcare systems.

Although the DRP has the potential to enrich training, it has also generated significant debate. Critics argue that the additional posting reduces valuable time from an already condensed three-year postgraduate curriculum and disrupts academic continuity. Others, however, view the program as a necessary reform that enhances patient exposure, addresses human resource shortages in secondary care hospitals, and bridges gaps between tertiary academic centers and the health services sector [2,3].

Despite ongoing discussions, empirical evidence on stakeholder satisfaction, operational barriers, and the program’s impact on postgraduate training remains scarce. Previous assessments have largely relied on administrative reports rather than systematically gathered stakeholder data, leaving a gap in understanding how the DRP functions at the ground level. Kerala provides a unique context for this evaluation due to its early statewide rollout, high health system maturity, and dual administrative control between the Directorate of Medical Education (DME) and the Directorate of Health Services (DHS). Insights from this context can offer transferable lessons for optimizing DRP implementation nationally.

To comprehensively explore this complex program, a mixed-method approach was adopted. The quantitative survey captured broad patterns of stakeholder perceptions, while the Delphi process facilitated expert consensus on key barriers and potential solutions-allowing both breadth and depth of understanding.

Kerala, a state with high literacy rates, strong public health indicators, and strict standards in medical education, was among the first in India to operationalize the DRP. With one full year of implementation completed, the state provides a unique setting to evaluate the successes and challenges of the program.

This study aims to examine the perceptions of stakeholders, including residents, the DME, and the DHS, regarding DRP implementation in Kerala. Using a mixed-methods approach that integrates quantitative and qualitative findings, we sought to identify the strengths and weaknesses of the program and to generate evidence-based insights that may inform its improvement both within the state and nationally. The objective is to assess stakeholder perceptions and identify systemic barriers and facilitators in the implementation of the DRP in Kerala, India, using a mixed-method design integrating quantitative survey and Delphi consensus techniques.

## Materials and methods

Study design and rationale

A mixed-method cross-sectional design was adopted, combining quantitative and qualitative approaches. This study employed a mixed-method design integrating quantitative and qualitative approaches. The quantitative component consisted of a cross-sectional survey conducted among postgraduate residents and officials from the DME and DHS in Kerala to assess their perceptions of the DRP. The qualitative component involved a series of iterative Delphi rounds among selected experts from both directorates to achieve consensus on key challenges and potential solutions related to DRP implementation. This design enabled triangulation of numerical trends with expert consensus to provide a comprehensive understanding of the program’s functioning.

Study setting and population

The study was conducted at Government Medical College, Thrissur, Kerala, with participation from stakeholders across the state. The study population comprised three groups: (i) postgraduate residents who had undergone DRP postings; (ii) officials from the DME, including heads of departments, professors, and institutional DRP nodal officers; and (iii) officials from the DHS, including superintendents, DRP nodal officers, and directors associated with DRP centers.

Sampling and recruitment

The quantitative survey component used convenience sampling to include the maximum possible number of postgraduate residents and health administrators actively involved in DRP implementation during the study period (January-June 2024). Based on DME records, approximately 720 postgraduate residents across all government medical colleges in Kerala were eligible for the DRP postings during this period. Of these, 282 residents responded to the survey (estimated response rate ≈39%). Among administrative stakeholders, approximately 260 DME officials and 190 DHS officials were eligible; responses were received from 180 DME and 124 DHS officials.

The qualitative Delphi component used purposive sampling to recruit experts with prior experience in DRP implementation or supervision, ensuring representation from both DME and DHS hierarchies. A total of 25 experts were invited, and 22 completed all three Delphi rounds (retention 88%).

The sample size was determined pragmatically, based on feasibility and expected response rates from prior statewide stakeholder surveys, rather than on formal statistical power calculation, since the study aimed for descriptive rather than inferential analysis.

We acknowledge that convenience and purposive sampling introduce potential selection bias and may limit generalizability. However, these approaches were necessary to capture diverse stakeholder views across Kerala within logistical constraints. To mitigate bias, participation invitations were disseminated through official DME and DHS communication channels statewide, and responses were accepted from all districts to ensure wide geographic representation.

Inclusion and exclusion criteria

All postgraduate residents who had completed at least one DRP posting in Kerala during the study period were eligible. For the DME group, the inclusion criteria were faculty members or nodal officers directly responsible for postgraduate training. For the DHS group, the inclusion criteria were senior officials working in DRP-linked centers. Exclusion criteria were non-consenting individuals and those without direct involvement in DRP implementation.

Questionnaire development and validation

The questionnaire was developed following a review of existing literature on postgraduate residency programs and consultation with subject experts in surgery, medicine, and community medicine (see Appendix). A draft version was piloted among 20 residents and 10 faculty members who were not included in the final study. Based on their feedback, minor modifications were made to improve clarity and relevance. Internal consistency was assessed using Cronbach’s alpha, which showed good reliability (α = 0.82). Content validity was established through expert review by a panel of five senior faculty members from clinical and paraclinical disciplines.

Delphi technique

The qualitative component employed a three-round Delphi technique to obtain consensus among experts from the DME and DHS regarding the challenges and facilitators in DRP implementation. Twenty-five experts were invited, and 22 completed all three rounds (retention 88%).

Round 1

Participants were asked to provide open-ended responses to broad questions such as “What are the major challenges faced in implementing the DRP at district hospitals?”, “What strategies have been effective in improving academic supervision?”, and “What structural or policy changes could enhance DRP outcomes?” Responses were thematically analyzed to generate an initial list of recurring issues and recommendations.

Round 2

The summarized list of themes from Round 1 (e.g., infrastructure adequacy, supervision quality, rotation planning, accommodation, monitoring mechanisms, interdepartmental coordination) was circulated to the same panel. Experts rated each theme for importance and feasibility on a five-point Likert scale (1 = not important/feasible to 5 = extremely important/feasible).

Round 3

Items that did not achieve consensus in Round 2 were re-circulated with anonymized feedback summaries. Participants were asked to re-rate these items to confirm or revise their earlier responses.

Consensus operationalization

Consensus was defined a priori as ≥70% agreement among respondents rating an item as “important” or “extremely important” (scores 4 or 5). Themes reaching this threshold were considered to have achieved consensus. Items failing to reach consensus after three rounds were documented but not included in the final priority list.

Thematic synthesis of the final Delphi findings was conducted using NVivo version 12, allowing integration with the quantitative survey results to identify convergent and divergent insights.

Data collection and analysis

Quantitative data were entered into Microsoft Excel (Microsoft Corp., USA) and analyzed using IBM SPSS Statistics for Windows, version 31.0 (released 2025, IBM Corp., Armonk, NY). Only descriptive statistics (frequencies, percentages, and means ± standard deviations) were applied, as the primary objective of this study was exploratory, to document stakeholder perceptions rather than to test hypotheses or compare predefined subgroups. Inferential statistical testing was therefore deemed inappropriate because the sampling strategy was non-random (convenience-based) and the dataset was not designed for inferential generalization.

Qualitative data from the Delphi rounds and open-ended survey responses were analyzed using thematic analysis in NVivo version 12. Two researchers independently coded the data using a hybrid framework that combined inductive and deductive approaches. The initial coding framework was guided by the study objectives and included domains such as infrastructure, supervision, academic coordination, workload, and administrative support. Additional codes were added inductively as new ideas emerged. Discrepancies between coders were resolved through discussion, and inter-rater reliability was assessed using Cohen’s kappa (κ = 0.79), indicating substantial agreement.

The study followed a convergent mixed-method design, wherein quantitative survey results and qualitative Delphi findings were analyzed separately and then integrated during interpretation. Integration was achieved through a side-by-side comparison of results in a joint display matrix and narrative weaving in the Discussion section. Specifically, quantitative trends (e.g., proportion of residents reporting inadequate supervision or satisfaction with accommodation) were compared with corresponding qualitative themes (e.g., “variable faculty engagement,” “need for structured feedback”). Areas of convergence (e.g., recognition of DRP’s positive impact on clinical confidence) and divergence (e.g., differing perceptions of monitoring adequacy between administrators and residents) were identified to generate actionable insights on program improvement.

This integrative approach enhanced data validity by triangulating multiple perspectives and aligning stakeholder perceptions with empirical patterns observed in the survey data.

Examples of emergent themes included “limited operative exposure due to inadequate theatre scheduling,” “variation in supervision across departments,” “need for joint DME-DHS monitoring,” and “enhanced clinical confidence after DRP posting.” These themes were then synthesized with quantitative findings to identify convergent and complementary insights.

Ethical approval for the study was obtained from the Institutional Ethics Committee, Government Medical College, Thrissur (IEC/GMCTSR/2024/242). Informed consent was obtained from all participants prior to data collection. Since no personal or sensitive data were disclosed, only aggregated findings are presented in the public domain.

## Results

A total of 586 stakeholders participated in the study, comprising 282 (48.1%) junior residents, 180 (30.7%) officials from the DME, and 124 (21.2%) officials from the DHS. Among the DHS respondents, 46 (37.1%) were institutional DRP nodal officers, 24 (19.4%) were DRP coordinators, 41 (33.1%) were superintendents, four (3.2%) were deputy directors, five (4.0%) were additional directors, and two (1.6%) were program officers. From the DME side, 122 (67.8%) were heads of departments, 14 (7.8%) were professors, and 30 (16.7%) were institutional DRP nodal officers.

The mean age of the residents was 28.4 ± 2.1 years, with near-equal gender distribution. Most residents were in their second or third year of training. Their specialty distribution included general medicine (22%), general surgery (20%), pediatrics (12%), obstetrics and gynecology (10%), anesthesia (8%), orthopedics (7%), radiology (6%), emergency medicine (5%), pathology (5%), and others (5%). Among DME officials, the mean duration of service was 14.2 ± 5.6 years, while DHS officials reported an average of 17.8 ± 7.2 years of service.

**Figure 1:**
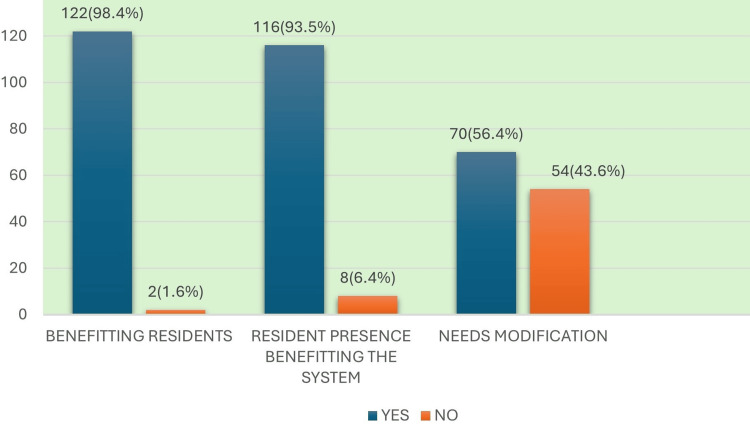
District Residency Program (DRP)’s contribution to improved training/academics/hospital work functioning Image created by the authors with MS Excel (Microsoft Corp., USA)

Feedback from DHS officials showed that the DRP was generally well-received at the district level. Most respondents felt the program improved hospital functioning and postgraduate training, while a notable minority pointed to delays in issuing completion certificates and uneven resident distribution across districts. These perspectives include DRP contributions, overall impressions, state-level coordination, and residents’ cooperation. DHS officials reported that the DRP contributed positively to hospital functioning and postgraduate academic training, as illustrated in Figure 1.

**Figure 2:**
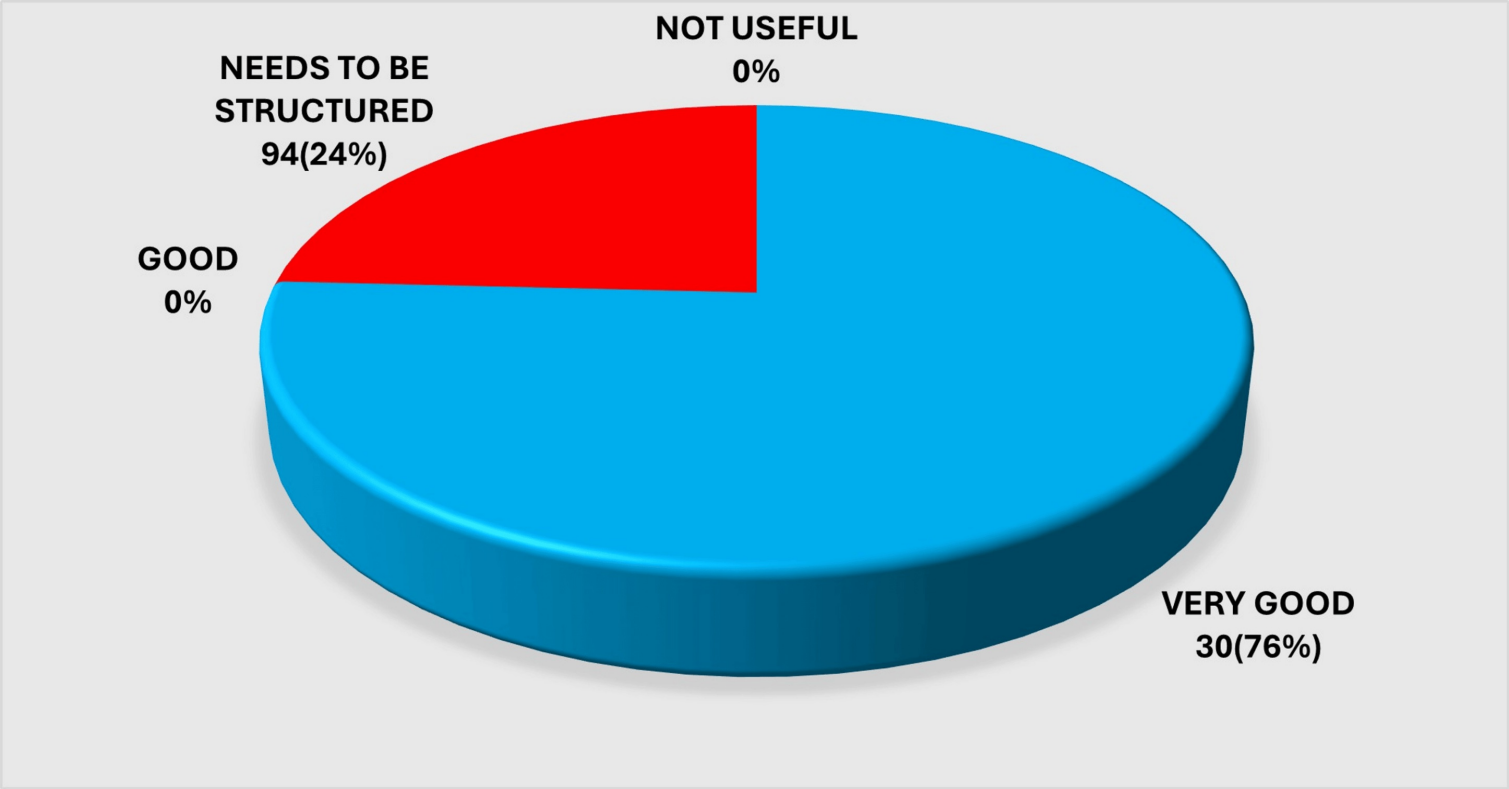
Impression regarding the District Residency Program (DRP) Image created by the authors with MS Excel (Microsoft Corp., USA)

When asked for an overall appraisal of the program, most DHS respondents expressed a favorable impression of DRP implementation, while a smaller group remained neutral or noted operational difficulties. This snapshot of general sentiment toward the program is summarized in Figure 2.

**Figure 3:**
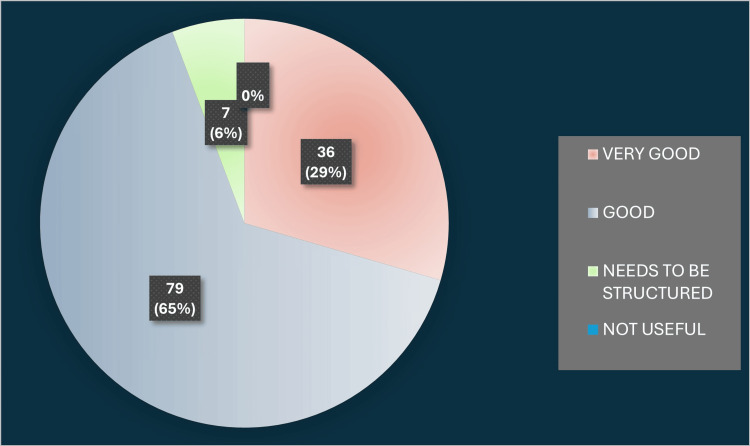
State coordination Image created by the authors with MS Excel (Microsoft Corp., USA)

State-level coordination was another key metric assessed by DHS officials. Over two-thirds felt that the communication, scheduling, and administrative oversight provided by the state were good or excellent, although a minority cited delays in certificate issuance. Their detailed ratings of state coordination appear in Figure 3.

**Figure 4:**
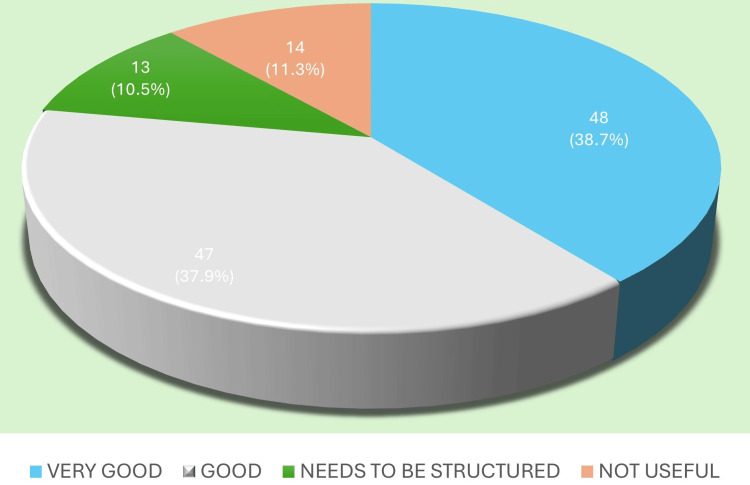
Resident cooperation Image created by the authors with MS Excel (Microsoft Corp., USA)

Cooperation from postgraduate residents was regarded as high, with many DHS respondents commending the residents’ professionalism and willingness to adapt to district-level demands. These perceptions of resident cooperation are presented in Figure 4.

**Figure 5:**
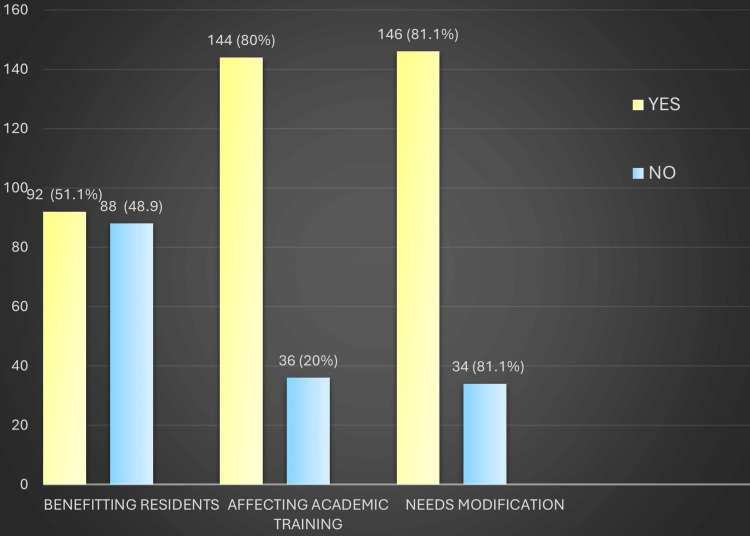
District Residency Program (DRP)’s contribution to improved training/academics Image created by the authors with MS Excel (Microsoft Corp., USA)

**Figure 6:**
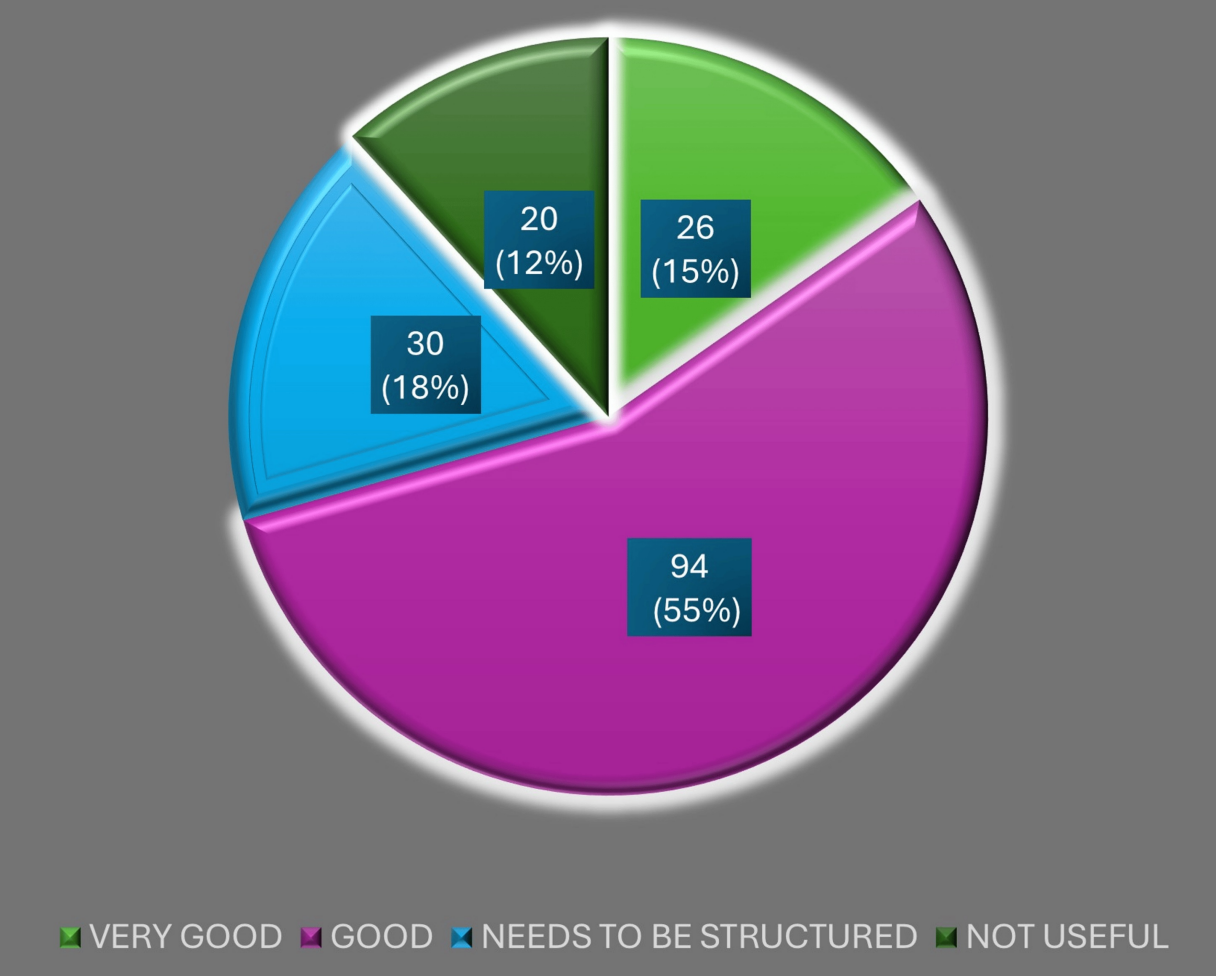
Overall impression of the District Residency Program (DRP) as per the Directorate of Medical Education (DME) Image created by the authors with MS Excel (Microsoft Corp., USA)

**Figure 7:**
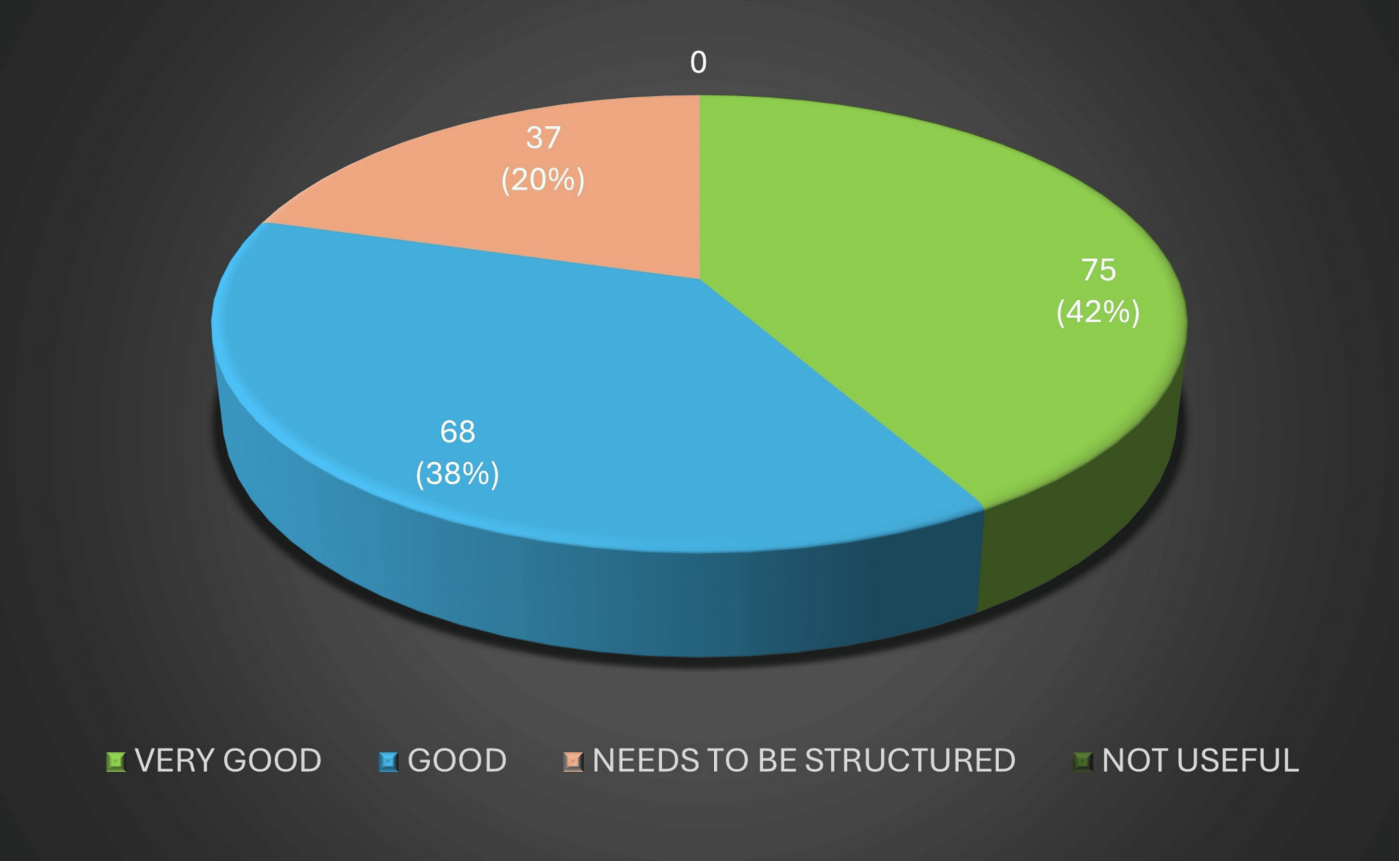
State coordination Image created by the authors with MS Excel (Microsoft Corp., USA)

**Figure 8:**
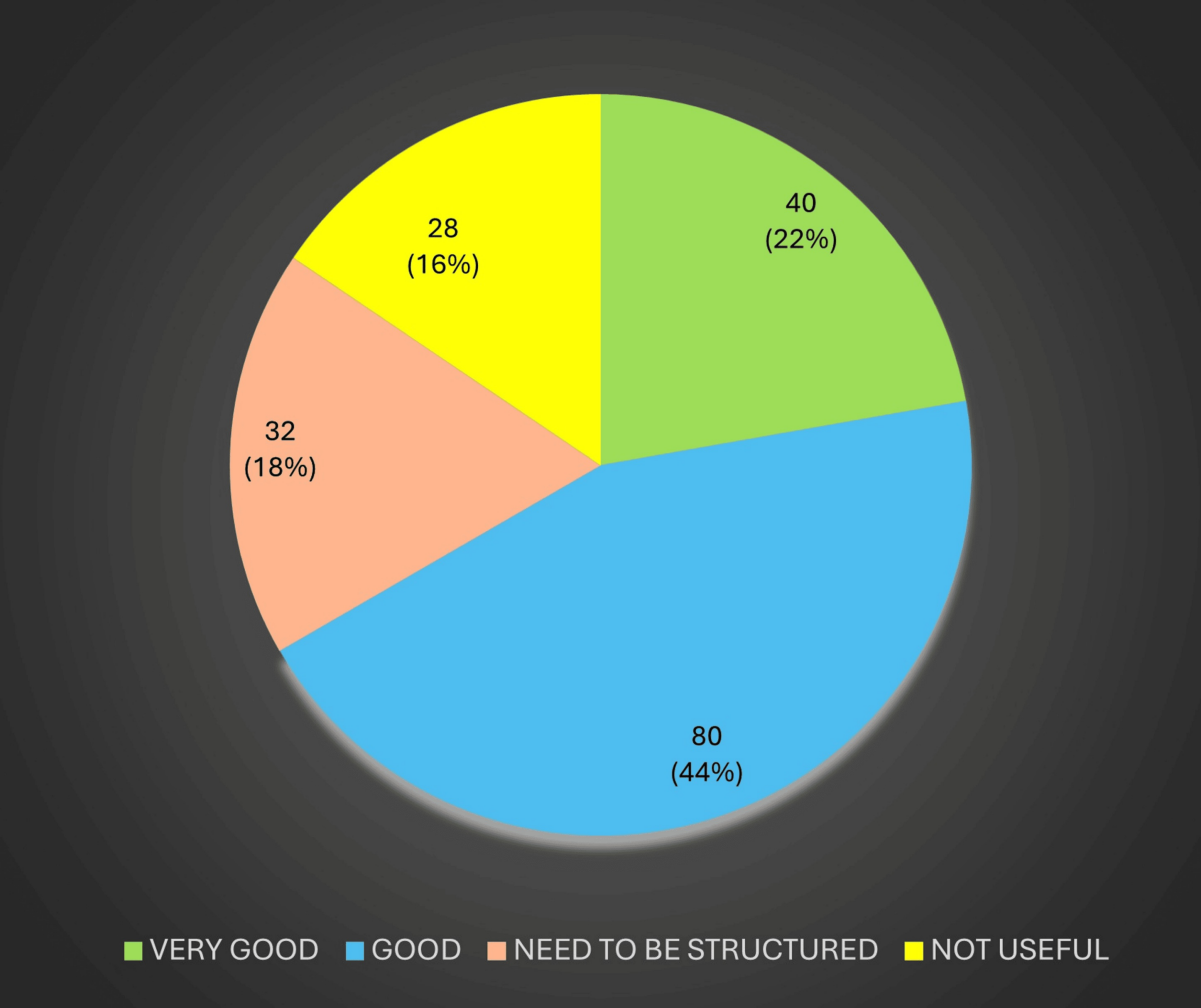
Impression regarding institutions under the Directorate of Health Services (DHS) (District Residency Program (DRP) centres) Image created by the authors with MS Excel (Microsoft Corp., USA)

Feedback from the DME side is depicted in Figures 5-8, which reflect opinions regarding the DRP’s impact on postgraduate training, overall impressions, coordination at the state level, and the functioning of DHS institutions hosting DRP residents.

DME officials’ views on the DRP’s contribution to postgraduate training and hospital academic activities are presented in Figure 5.

DME respondents also provided an overall judgment of the DRP’s success. Most rated the initiative positively, while a few pointed to challenges such as limited specialty-specific supervision and uneven resource allocation. These overall impressions are depicted in Figure 6.

Regarding state-level coordination, DME officials reported predominantly good to excellent experiences, citing efficient communication with the Directorate of Health Services and timely administrative support. The distribution of these assessments is illustrated in Figure 7.

DME participants further evaluated the performance of DHS institutions that hosted the residents, noting areas of strength like patient volume as well as gaps in specialty coverage and monitoring. Their collective views on the functioning of DRP centers under DHS are presented in Figure 8.

**Figure 9:**
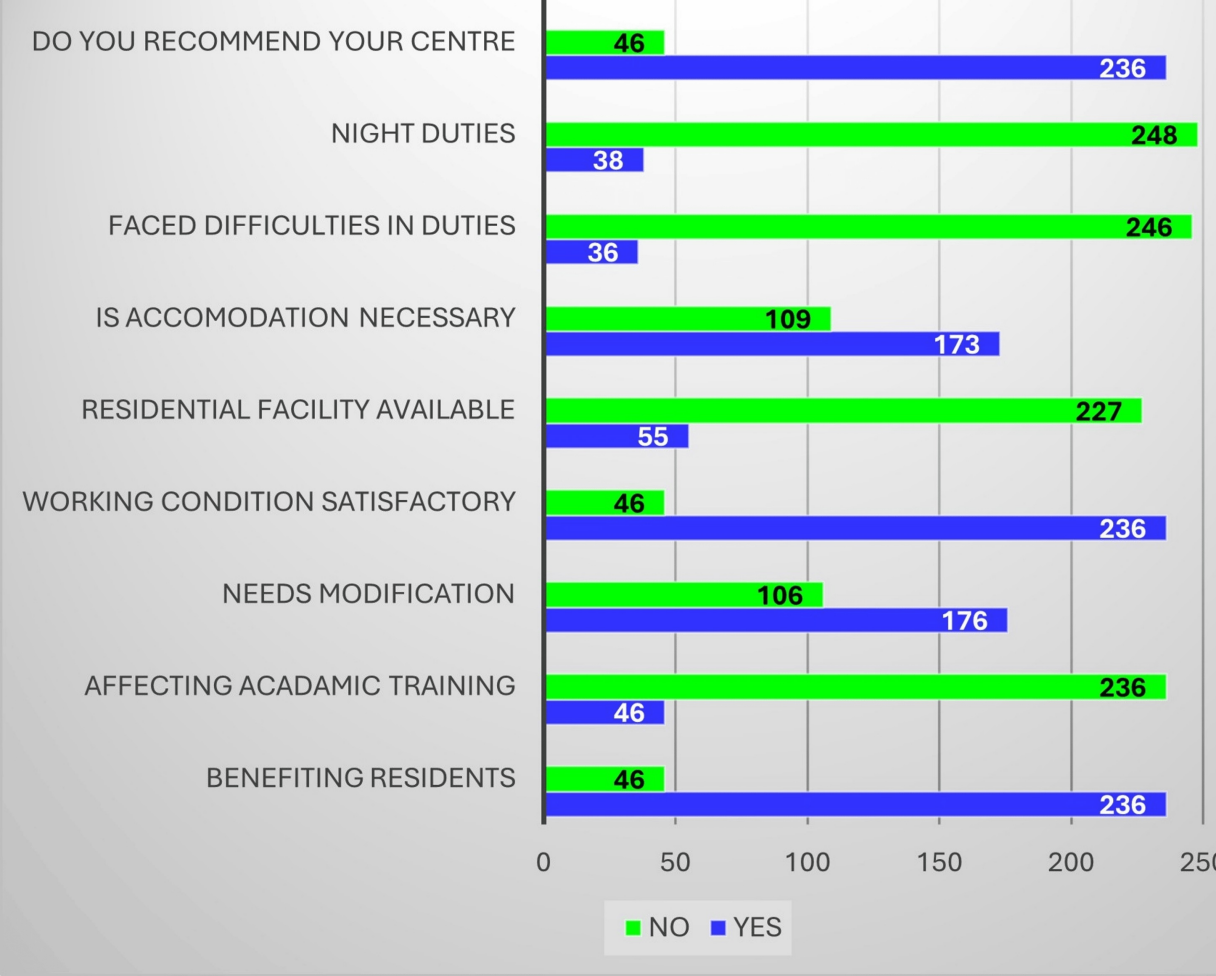
Junior resident feedback about the pros and cons of the District Residency Program (DRP) Image created by the authors with MS Excel (Microsoft Corp., USA)

**Figure 10:**
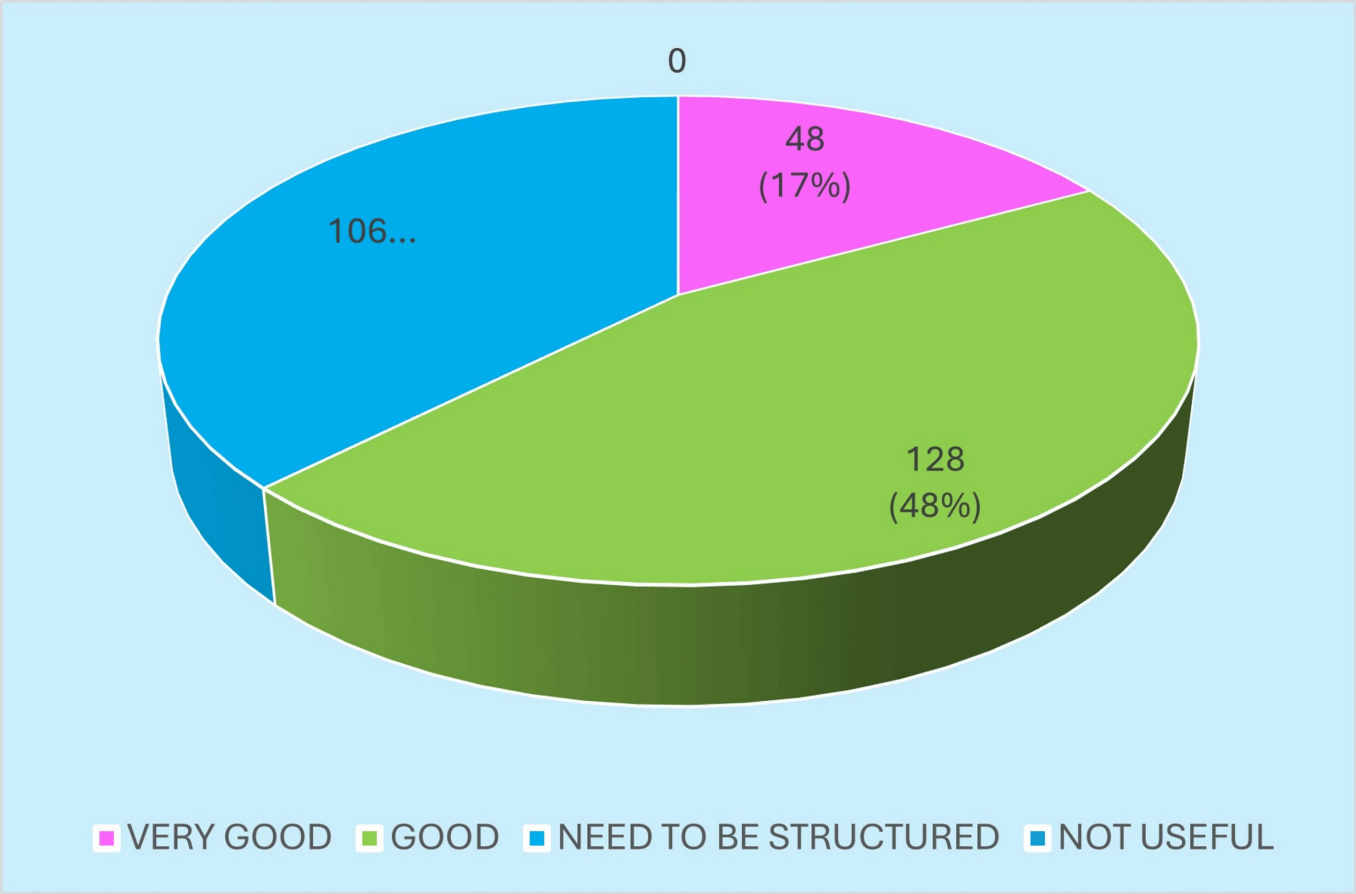
Impression regarding the District Residency Program (DRP) Image created by the authors with MS Excel (Microsoft Corp., USA)

**Figure 11:**
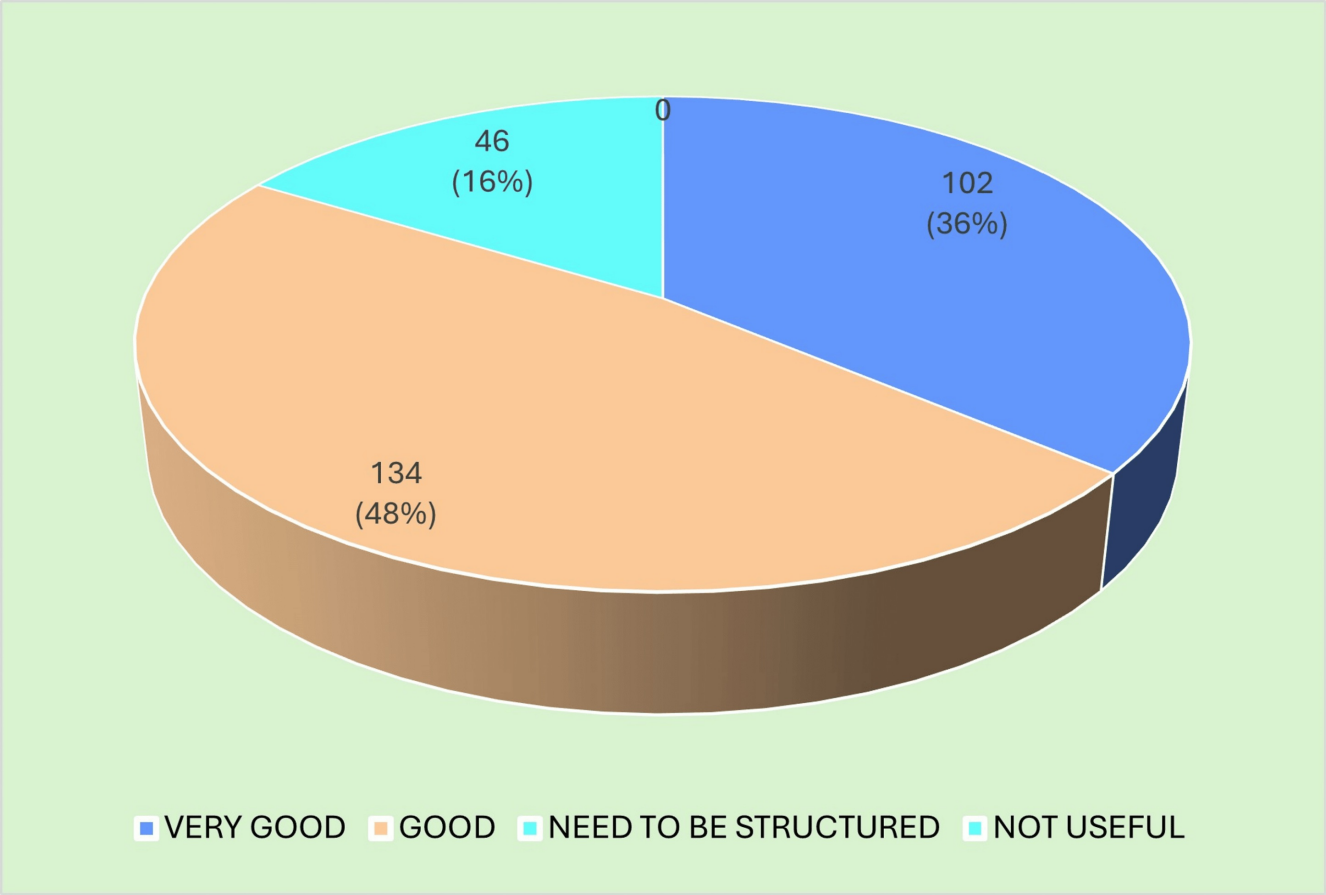
State coordination Image created by the authors with MS Excel (Microsoft Corp., USA)

**Figure 12:**
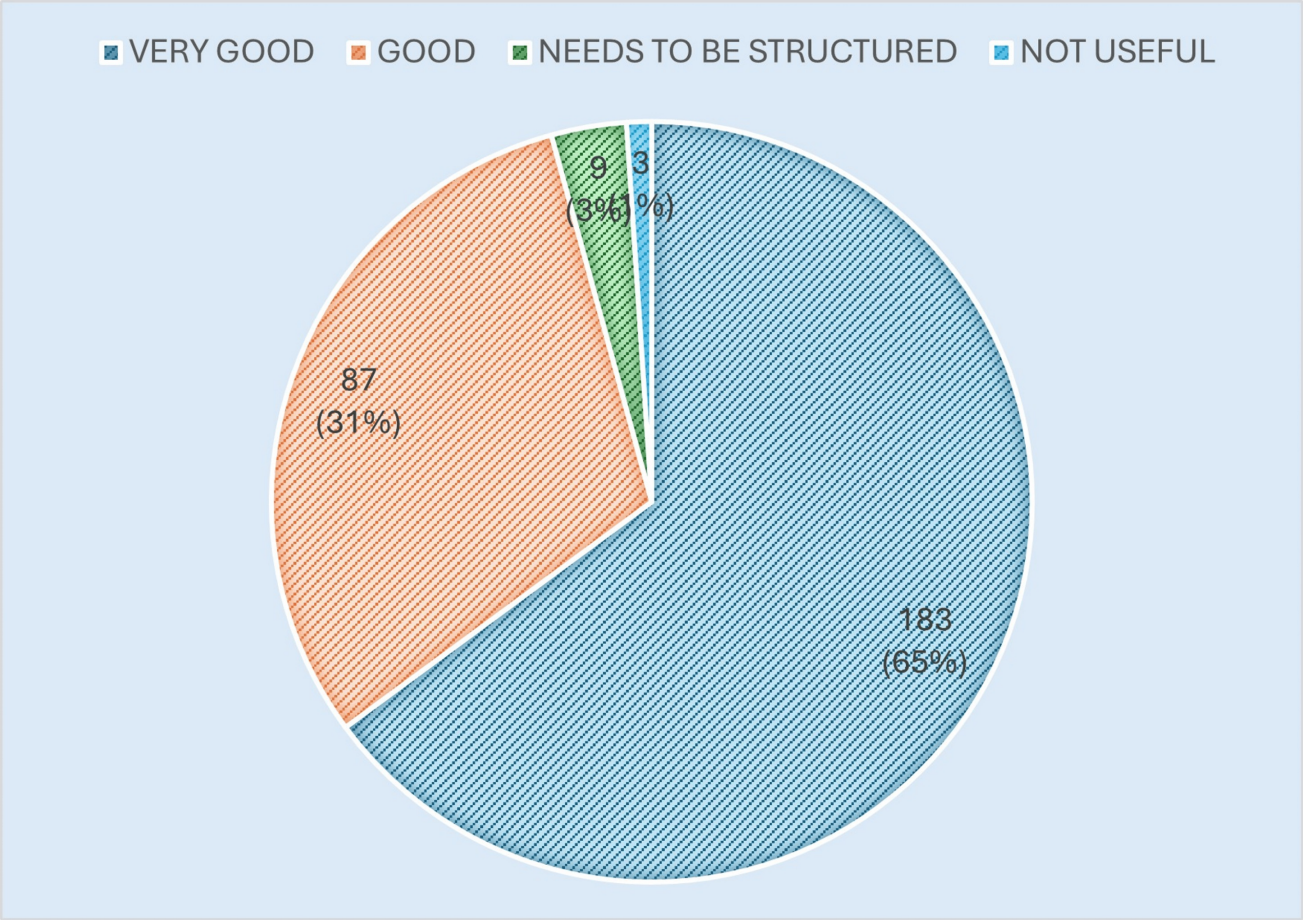
Impression regarding institutions under the Directorate of Health Services (DHS) (District Residency Program (DRP) centres) Image created by the authors with MS Excel (Microsoft Corp., USA)

Junior resident perspectives are presented in Figures 9-12, summarizing their views on the advantages and challenges of DRP, overall impressions of the program, coordination, and experiences at DHS institutions. Junior residents’ feedback highlighting the main advantages and challenges of the DRP is depicted in Figure 9.

When summarizing their overall experience, a majority of residents reported satisfaction with the DRP despite the logistical and academic hurdles described above. This overall impression of the program is illustrated in Figure 10.

Residents also commented on the efficiency of state-level coordination, with most agreeing that scheduling, communication, and problem resolution were well managed. Their ratings of coordination quality are summarized in Figure 11.

Finally, residents assessed the DHS institutions where they were posted, discussing factors such as infrastructure, supervision, and staff cooperation. These collective impressions of district centres are presented in Figure 12.

Qualitative insights (Delphi findings) and Delphi analysis (n = 25 participants; 88% retention across three rounds) yielded the following key themes: 1) Structured academic support: The consensus of 20/25 (80%) recommended specialty-specific postings and structured logbooks to ensure adequate training. 2) Monitoring and evaluation: The consensus of 22/25 (88%) suggested unit-level and state-level assessment frameworks to track resident performance and center quality. 3) Infrastructure and logistical support: The consensus of 21/25 (84%) emphasized residential facilities or transport allowances to reduce travel fatigue. 4) Duration and timing of DRP: The consensus of 18/25 (72%) recommended reducing DRP duration to one to two months to minimize disruption to academic schedules. Illustrative quotes include “Without a radiology consultant at the center, residents cannot meaningfully interpret scans.” “Travel expenses consume a significant portion of our stipend, limiting engagement in hospital activities.” In the cross-stakeholder synthesis convergence, both residents and administrators recognized the need for improved supervision, structured academic activities, and equitable postings. For divergence, while administrators rated coordination highly, residents highlighted practical challenges such as travel fatigue, accommodation, and being used for service coverage rather than training. For the integration of mixed methods, quantitative satisfaction rates were supported by qualitative themes, revealing that positive overall ratings co-existed with targeted operational concerns.

System of implementation

Stakeholders reported that the overall coordination of the DRP was satisfactory. Among DHS respondents, 72% rated state-level coordination as good or excellent, while 69% of DME officials expressed similar views. However, challenges in implementation were identified. Over half (54%) of DHS respondents noted delays in issuing completion certificates, and 46% highlighted uneven distribution of residents across districts, with some areas overcrowded and others, such as Wayanad, underserved. Qualitative findings supported these observations. DHS officials emphasized the need for a structured evaluation and monitoring system and recommended equitable posting of residents across districts. One respondent noted, “Some districts have far too many residents, while others struggle with shortages. This creates an imbalance in service delivery.”

Academic impact

Most residents (84%) reported that the DRP did not significantly disrupt their academic training and that working conditions were generally satisfactory. Despite overall satisfaction with the DRP, 41% of residents reported reduced opportunities for structured teaching activities. Faculty respondents, especially from the DME, corroborated this, noting limited hands-on exposure in certain specialties such as radiology, preclinical, and paraclinical departments. Residents frequently expressed that postings in centers lacking specialty-specific consultants constrained their educational experience. These findings underscore the need for structured academic activities, specialty-aligned postings, and supervised clinical engagement to enhance the training value of the DRP. For example, radiology residents reported inadequate training opportunities in centers without qualified consultants, while preclinical and paraclinical residents noted that their laboratory-based learning needs were not met.

Integration of qualitative data highlighted similar concerns. Residents frequently expressed that postings in specialties without corresponding consultants limited the educational value of their training. As one radiology resident explained, “Without a radiology consultant at the center, we could not perform ultrasounds or meaningfully interpret scans.” Faculty echoed this sentiment, recommending specialty-specific postings and structured logbooks to monitor academic activity.

Overall satisfaction and infrastructure

Despite challenges, stakeholders broadly acknowledged the positive impact of DRP. Seventy-six percent of DME officials and 69% of DHS officials agreed that the DRP contributed positively to postgraduate training (Figures 5-6). Among residents, 61% emphasized the need for accommodation facilities (Figure 9), and 58% reported financial strain due to daily travel costs averaging INR 4,000 per month.

Qualitative feedback triangulated these concerns. Residents repeatedly called for residential facilities or, at a minimum, transportation allowances. One resident remarked, “It is very difficult to travel every day, both physically and financially. Travel expenses consume a significant portion of our stipend.” Others noted that inadequate accommodation limited their ability to participate fully in hospital activities.

Concerns were also raised about residents being treated as substitutes for medical officers. Several respondents reported disproportionate workloads compared with in-house DNB trainees, stating that the original intent of the DRP as a training program was diluted when residents were deployed primarily for service coverage. A resident expressed this sentiment as, “DRP centers should be training institutions, not just places where PGs are used to cover staff shortages."

From the DME respondents, several themes emerged. Pathology residents from private medical colleges were suggested to be posted in district hospitals for autopsy training and in state or regional public health laboratories for laboratory exposure. Concerns were expressed about the limited hands-on experience of radiology residents due to the lack of qualified radiology consultants in DHS centers, while preclinical and paraclinical trainees were noted to require more laboratory-based training. The need for a structured monitoring system was frequently emphasized, with suggestions for implementing a mentor-mentee framework, maintaining logbooks, recording attendance, preparing detailed activity schedules, and conducting end-of-posting assessments. One respondent remarked that the DRP at times resembled an “extended holiday” owing to the absence of structured supervision. A minority also suggested that the DRP might be more effectively implemented as a mandatory program after completion of postgraduate training, rather than during residency.

Among DHS officials, a recurring concern was the lack of initiative from residents, with some respondents perceiving limited engagement and empathy in patient care. Inequitable distribution of residents across districts was also highlighted, with some districts experiencing overcrowding while others, such as Wayanad, received very few residents. DHS respondents emphasized the importance of a formal evaluation and feedback mechanism, with unit chiefs providing structured assessments of residents, and raised concerns about delays in issuing completion certificates. Many also called for better accommodation arrangements, such as access to government rest houses, and suggested extending the DRP model to include Diplomate of National Board (DNB) trainees.

Resident feedback revealed several important themes. Many called for a reduction in the duration of DRP postings, particularly for non-clinical disciplines such as Anatomy, Physiology, and Pharmacology, where they felt that limited benefit was derived. Residents repeatedly emphasized the need to be posted under consultants from their own specialties, with radiology residents requesting postings in centers with qualified radiology consultants to allow meaningful exposure to imaging, and emergency medicine residents preferring placements in higher-level centers with adequate facilities. Residents from laboratory-based specialties suggested that they should be placed in centers with active laboratory services, while several respondents requested that the duration of postings in public health laboratories be increased to two months.

Concerns regarding academic and research opportunities were widespread. Many residents reported that DRP adversely affected their academic training, and some suggested canceling the program or moving it to the end of the postgraduate course. Others pointed out the potential for research using health data available at the district level, which they felt was underutilized. Accommodation and travel were recurring challenges, with many residents reporting high travel expenses, averaging around INR 4,000 per month, and requesting provision of travel allowances or pooled transport facilities.

Residents also highlighted the need for clear duty guidelines, timely attendance reporting to avoid stipend delays, and specialty-specific digital logbooks. Many expressed dissatisfaction with being used to compensate for staff shortages, reporting that they were often burdened with additional duties compared to in-house DNB residents. Several noted that the original intent of DRP - as a structured training program - was diluted when residents were instead used as substitutes for medical officers. Finally, residents drew attention to workplace dynamics, with some reporting that interactions with other staff could be improved, remarking that “we are residents who came there to help.”

Overall, the analysis of responses across all stakeholder groups provides a comprehensive picture of the strengths and challenges of DRP implementation in Kerala, while also offering actionable suggestions for its improvement.

## Discussion

The findings of this study highlight both the strengths and challenges of the DRP in Kerala, echoing concerns reported in other parts of India. A study from Rajasthan found that only 17% of residents felt the learning objectives of the DRP were fulfilled, while nearly 60% reported feeling isolated from their academic departments. More than half were not posted within their own specialties, 80% expressed concerns about safety, and over 75% were dissatisfied with basic amenities [4]. Similarly, a study from Moradabad identified strengths such as enhanced patient exposure for residents from private institutions and support for peripheral human resource needs, but also weaknesses, including residents replacing medical officers, inadequate orientation, insufficient resources, and perceptions of postings as “leisure assignments” [5].

These concerns resonate with our findings in Kerala. Residents frequently reported inadequate specialty-specific training opportunities and highlighted the need for structured academic activities during postings. Officials from the DME and DHS confirmed these gaps, emphasizing inequitable distribution of residents across districts and insufficient monitoring mechanisms. Previous literature also points to additional challenges, including reduced time for thesis work, disruption of academic schedules, transport difficulties, and inadequate amenities at district centers [6]. The consistency of these themes across studies suggests that the challenges associated with DRP implementation are widespread and systemic rather than isolated to a single state.

Our study strengthens the evidence base by incorporating perspectives from multiple stakeholder groups, rather than focusing solely on residents. The combination of quantitative surveys and qualitative Delphi analysis allowed triangulation of findings, providing both measurable trends and nuanced insights. For example, while 61% of residents indicated that accommodation was necessary, qualitative responses elaborated on the financial strain and travel fatigue associated with daily commuting. Similarly, although more than two-thirds of officials rated state-level coordination positively, open-ended feedback revealed frustrations with delays in certificate issuance and inconsistent supervision. This mixed-method approach thus provided a more comprehensive and balanced view of the DRP’s current status.

Analytical depth: understanding the findings

Reduced structured teaching (41% of residents) linked to the absence of specialty-specific consultants and variability in DHS institutional resources.

Positive overall satisfaction may reflect strong state-level coordination and resident adaptability, but resource constraints and uneven supervision explain residual dissatisfaction.

Discrepancies between DME and DHS perceptions (e.g., monitoring adequacy, academic engagement) highlight institutional communication gaps.

Integration of mixed-method findings

Quantitative satisfaction rates (e.g., 84% reporting satisfactory working conditions) align with the Delphi consensus that residents are generally cooperative and that DRP improves hospital functioning.

Qualitative insights add nuance: despite overall satisfaction, logistical issues, accommodation, and specialty-specific gaps limit training quality.

Contextualization: Kerala’s unique setting

High literacy, robust public health system, and effective state-level coordination may explain higher satisfaction rates compared to Rajasthan and Moradabad.

Kerala’s district hospitals have better staffing ratios and infrastructure, yet challenges persist in specialty coverage and supervision, underscoring structural limits even in well-resourced states.

Recommendations linked to evidence

Administrative recommendations include streamlining certificate issuance, implementing structured monitoring, and equitably distributing residents (supported by DHS feedback on delays and overcrowding).

Academic recommendations include specialty-aligned postings, structured teaching sessions, mentor-mentee framework, and digital logbooks (supported by residents and DME faculty reporting limited hands-on exposure).

Infrastructural recommendations are provision of accommodation, transportation allowances, and improved laboratory and clinical facilities (supported by residents’ qualitative feedback on travel burden and lack of resources).

Cautious interpretation

Claims about generalizability or evidence contribution are tempered, acknowledging convenience/purposive sampling and descriptive study design.

Findings provide state-level insights for Kerala while offering a framework for other states to adapt, rather than claiming national generalizability.

Several recommendations emerged from the study. Many stakeholders felt that the duration of the DRP could be reduced to one or at most two months to limit disruption of postgraduate academics. It was widely agreed that specialty-specific learning objectives should be clearly defined and standardized at the national level, ideally through a committee constituted by the National Medical Commission. The preparation of structured modules and mandatory orientation for district-level doctors were suggested as means to improve uniformity in training. Respondents also recommended the introduction of digital logbooks and a centralized certification system to improve accountability and streamline documentation.

Infrastructure and support systems were also highlighted as essential areas for improvement. Provision of residential facilities at DRP centers, or alternatively transportation allowances, was strongly recommended by both residents and administrators. Stakeholders also suggested periodic appraisal of DRP centers by a state-level team, with the authority to include or exclude centers based on compliance with training standards. Finally, several respondents proposed extending DRP to DNB trainees, noting that the program could strengthen their training while also enhancing the health system.

By addressing these challenges and implementing targeted reforms, the DRP could evolve into a more structured and impactful program that balances service delivery with postgraduate education. In its current form, the program demonstrates promise but requires adjustments to fully realize its dual goals of strengthening health services and enriching resident training.

Limitations and future directions

This study has several limitations. The use of convenience and purposive sampling may limit representativeness, and reliance on self-reported perceptions introduces the possibility of social desirability bias. Only descriptive statistics were applied, precluding inferential testing or hypothesis-driven comparisons. Despite these limitations, the integration of quantitative and Delphi-derived qualitative data provides a detailed snapshot of stakeholder experiences. Future research should include longitudinal evaluation of DRP outcomes, including residents’ skill acquisition, clinical competence, and patient care metrics. In addition, Delphi feedback can inform structured monitoring frameworks, enabling systematic assessment of training quality, specialty-specific supervision, and administrative coordination in district hospitals. Comparative studies across states may further identify best practices and guide national-level DRP optimization.

## Conclusions

The DRP in Kerala demonstrates strong state-level coordination and resident cooperation, yet implementation would benefit from systematized monitoring and equitable resident distribution to address observed gaps in supervision and certificate issuance. While clinical residents generally maintained satisfactory academic engagement, non-clinical and specialty-specific postings experienced inconsistent integration of structured learning opportunities, highlighting the need for targeted academic frameworks and mentor-guided activities. Finally, infrastructural and logistical improvements, including provision of accommodation, transportation support, and enhanced specialty-specific supervision, are essential to ensure sustainability and optimize both training quality and service delivery in district hospitals.

## Appendices

A structured questionnaire was developed to capture stakeholder perceptions of DRP implementation. It included sections on (i) general information (stakeholder category, specialty, experience), (ii) perceptions of DRP (contribution to training, impact on academic work, state-level coordination, resident cooperation), (iii) implementation challenges (infrastructure, supervision, workload, monitoring), and (iv) suggestions for improvement (duration, specialty-specific postings, inclusion of DNB trainees). Questions were a mix of Likert-scale, multiple-choice, and open-ended formats.

Target respondents: postgraduate students, faculty members, and administrators involved in the DRP

District Residency Programme: stakeholder perception questionnaire

Section A: Demographic Profile

1. Name (optional): ___________________________ 2. Designation: ☐ Postgraduate Student ☐ Faculty ☐ Head of Department ☐ Administrator 3. Specialty: ___________________________ 4. Institution Type: ☐ Government Medical College ☐ District Medical Office 5. Years of postgraduate teaching experience: ☐ <5 years ☐ 5-10 years ☐ >10 years

Section B: Awareness and Understanding

1. I am aware of the objectives of the District Residency Programme. ☐ Strongly Agree ☐ Agree ☐ Neutral ☐ Disagree ☐ Strongly Disagree 2. The DRP was adequately introduced and explained to all stakeholders before implementation. ☐ Strongly Agree ☐ Agree ☐ Neutral ☐ Disagree ☐ Strongly Disagree 3. I understand the expected outcomes and competencies to be gained during the DRP posting. ☐ Strongly Agree ☐ Agree ☐ Neutral ☐ Disagree ☐ Strongly Disagree

Section C: Implementation Experience

4. The process of posting residents to district hospitals was smooth and well coordinated. ☐ Strongly Agree ☐ Agree ☐ Neutral ☐ Disagree ☐ Strongly Disagree 5. The infrastructure and facilities in district hospitals were adequate to support residency training. ☐ Strongly Agree ☐ Agree ☐ Neutral ☐ Disagree ☐ Strongly Disagree 6. Faculty supervision and mentorship at district hospitals were satisfactory. ☐ Strongly Agree ☐ Agree ☐ Neutral ☐ Disagree ☐ Strongly Disagree 7. There was effective communication between parent institutions and district hospitals. ☐ Strongly Agree ☐ Agree ☐ Neutral ☐ Disagree ☐ Strongly Disagree 8. The duration of the district posting (3 months) was appropriate. ☐ Strongly Agree ☐ Agree ☐ Neutral ☐ Disagree ☐ Strongly Disagree

Section D: Perceived Benefits

9. The DRP improved the residents’ exposure to secondary-level healthcare. ☐ Strongly Agree ☐ Agree ☐ Neutral ☐ Disagree ☐ Strongly Disagree 10. The program enhanced residents’ clinical decision-making and independence. ☐ Strongly Agree ☐ Agree ☐ Neutral ☐ Disagree ☐ Strongly Disagree 11. The DRP strengthened teamwork and community-oriented learning. ☐ Strongly Agree ☐ Agree ☐ Neutral ☐ Disagree ☐ Strongly Disagree 12. The program contributed to improved healthcare delivery at district hospitals. ☐ Strongly Agree ☐ Agree ☐ Neutral ☐ Disagree ☐ Strongly Disagree

Section E: Challenges Faced

13. Lack of basic facilities or equipment affected the quality of training. ☐ Strongly Agree ☐ Agree ☐ Neutral ☐ Disagree ☐ Strongly Disagree 14. Accommodation and logistics were satisfactory during the district posting. ☐ Strongly Agree ☐ Agree ☐ Neutral ☐ Disagree ☐ Strongly Disagree 15. Communication gaps existed between district hospital and parent medical college. ☐ Strongly Agree ☐ Agree ☐ Neutral ☐ Disagree ☐ Strongly Disagree 16. Workload at district hospitals was manageable and appropriate for training purposes. ☐ Strongly Agree ☐ Agree ☐ Neutral ☐ Disagree ☐ Strongly Disagree

Section F: Suggestions and Way Forward (Qualitative Section)

1. What were the most positive aspects of the District Residency Programme in your experience? 2. What were the major challenges you encountered during DRP implementation? 3. How do you think faculty and institutional support can be improved? 4. In your opinion, how can the DRP posting be made more beneficial for postgraduate residents? 5. Any additional comments or suggestions for policy-level improvement:

Section G: Overall Perception

17. Overall, I consider the DRP a valuable component of postgraduate training. ☐ Strongly Agree ☐ Agree ☐ Neutral ☐ Disagree ☐ Strongly Disagree 18. I would recommend continuation of the DRP with necessary modifications. ☐ Strongly Agree ☐ Agree ☐ Neutral ☐ Disagree ☐ Strongly Disagree
